# Clinical Case of Mild Tatton–Brown–Rahman Syndrome Caused by a Nonsense Variant in *DNMT3A* Gene

**DOI:** 10.3390/clinpract14030073

**Published:** 2024-05-21

**Authors:** Fatima Bostanova, Olga Levchenko, Margarita Sharova, Natalia Semenova

**Affiliations:** Research Centre for Medical Genetics, 115522 Moscow, Russia

**Keywords:** Tatton–Brown–Rahman syndrome, *DNMT3A*, acute myeloid leukemia, intellectual disability, macrocephaly

## Abstract

Tatton–Brown–Rahman syndrome is a rare autosomal dominant hereditary disease caused by pathogenic variants in the *DNMT3A* gene, which is an important participant in epigenetic regulation, especially during embryonic development, and is highly expressed in all tissues. The main features of the syndrome are high growth, macrocephaly, intellectual disability, and facial dysmorphic features. We present a clinical case of Tatton–Brown–Rahman syndrome in a ten-year-old boy with macrocephaly with learning difficulties, progressive eye impairment, and fatigue suspected by a deep learning-based diagnosis assistance system, Face2Gene. The proband underwent whole-exome sequencing, which revealed a recurrent nonsense variant in the 12th exon of the *DNMT3A*, leading to the formation of a premature stop codon—NM_022552.5:c.1443C>A (p.Tyr481Ter), in a heterozygous state. This variant was not found in parents, confirming its de novo status. The patient case described here contributes to the understanding of the clinical diversity of Tatton–Brown–Raman syndrome with a mild clinical presentation that expands the phenotypic spectrum of the syndrome. We report the first recurrent nonsense variant in the *DNMT3A* gene, suggesting a mutational hot-spot. Differential diagnoses of this syndrome with Sotos syndrome, Weaver syndrome, and Cowden syndrome, as well as molecular confirmation, are extremely important, since the presence of certain types of pathogenic variants in the *DNMT3A* gene significantly increases the risk of developing acute myeloid leukemia.

## 1. Introduction

Tatton–Brown–Rahman syndrome (TBRS) is caused by a heterozygous causative variant in the *DNMT3A* gene [1]. Its hallmark clinical features include postnatal macrosomia, intellectual disability, and distinct facial dysmorphisms. Its expected prevalence is <1/1,000,000. The gene’s protein product, DNA methyltransferase 3A, operates in DNA modification via methylation [2].

Our understanding of the pathogenesis of this syndrome remains incomplete. In 2018, Tatton–Brown et al. published an article presenting a comprehensive characterization of 55 TBRS patients, revealing consistent phenotypic traits, including the following: tall stature, macrocephaly, dense horizontal eyebrows, narrow eye slits, an elongated oval face, a high-arched palate, and varying degrees of intellectual disability. Some patients also displayed additional features with variable frequency, such as mitral and tricuspid valve defects, atrial septal defects, pronounced joint hypermobility, and umbilical hernia [3,4].

In 2017, the first documented case of TBRS associated with the p.Arg882Cys missense substitution was reported. The patient developed acute myeloid leukemia (AML) at age 15 [5]. Notably, this single-nucleotide variant (SNV) also stands as the most common somatic mutation in AML [6,7,8,9]. Concurrently, Shen et al. established the association of eight additional SNVs found in TBRS with AML [10].

Notably, beyond its association with TBRS and AML, germline mutations within the *DNMT3A* gene could lead to Gaine–Spratou–Jackson syndrome (GSJS), characterized by a clinical phenotype. In stark contrast to TBRS, GSJS manifests with features such as dwarfism, low weight, microcephaly, and intellectual disability. Gaine et al., in their study, not only provided clinical descriptions of patients but also conducted a functional analysis of identified missense variants (p.Trp330Arg and Asp333Asn). Their investigation revealed an association between these variants and hypermethylation across diverse genomic regions, classifying them as gain-of-function (GoF) variants [11]. Considering the presence of both loss-of-function variants and GoF in the TBRS gene, it is reasonable to hypothesize that the variant’s impact on protein function contributes to the divergent phenotype. However, comprehensive investigations are essential to solidify and establish robust genotype–phenotype correlations.

## 2. Materials and Methods

Subjects: The clinical examination and genetic analysis of the proband was performed in the Research Centre for Medical Genetics, Russia. All research participants gave their informed consent to the clinical examination and publication of their anonymized data (for infant probands, the adult responsible for them signed a consent form). The study was performed in accordance with the Declaration of Helsinki and approved by the Institutional Review Board of the Research Centre for Medical Genetics, Russia. Written informed consent was obtained from the family.

Genetic analysis: The manuscript RefSeq accession numbers NG_029465.2, NM_022552.5, and NP_072046.2 were used for the *DNMT3A* gene.

Whole-exome sequencing (WES) of genomic DNA was performed on an Illumina NextSeq 500 instrument (Illumina, San Diego, USA) in 2 × 151 bp paired-end mode to an average depth of minimum 70×. The libraries were prepared and enriched using Illumina Nextera Rapid Capture Exome Kit v1.2.

Sanger sequencing was performed using the ABI PRISM Big Dye Terminator (v 3.1) Cycle Sequencing Kit (Applied Biosystems, Foster City, CA, USA) on the ABI3130xl Genetic Analyzer (Applied Biosystems, Foster City, CA, USA) according to the manufacturer’s recommendations.

## 3. Results

### 3.1. Case Description

The proband, a 10-year-old boy from a non-consanguineous family, received medical consultation in our center. The family pedigree was unremarkable, with two healthy elder sons. The proband was born from the mother’s third pregnancy with a birth weight of 4950 g (+2.83 SD), length of 60 cm (+3.75 SD), and an Apgar score of 8/9. Motor development occurred with minor temporal delays: he started to hold his head at 4 months, sat independently at 7 months, and walked independently at the age of 1 year and 3 months. Speech development initially matched age expectations, with a vocabulary of approximately ten words by the age of one. However, phrase speech emerged only at 3.5 years. The proband has been under the care of a neuropsychologist and a neurologist since the age of 3 years. Currently, he attends a specialized school with average academic performance.

Magnetic resonance imaging (MRI) of the brain conducted at the age of 4 revealed no signs of structural or focal pathology. Electroencephalography (EEG) showed no epileptiform activity. Echocardiography identified mitral and tricuspid valve prolapses. Progressive visual impairment was noted by the age of 8.5. Ophthalmological evaluation revealed partial atrophy of the optic nerves, secondary convergent strabismus, and simple myopic astigmatism in both eyes.

According to a medical psychologist consultation, the child exhibited a diminished intellectual capacity with immaturity and specific characteristics in the formation of the emotional–volitional sphere (according to the Wechsler scale: Verbal Intellectual Quotient (VIQ)—80, Non-Verbal Intellectual Quotient (NIQ)—85, Overall Intellectual Quotient (OI)—80). Speech–language pathology of a mild degree, dysgraphia, and elements of dyslexia were observed, leading to ongoing observation by a speech therapist due to systemic speech underdevelopment. There were no autism spectrum disorder features. Endocrine etiology for the child’s excessive growth was excluded through comprehensive examination (growth hormone: 6.4 ng/mL, IGF-1: 201 ng/mL, Prolactin: 259 mME/m). Also, he had no history of any oncological conditions.

**Figure 1:**
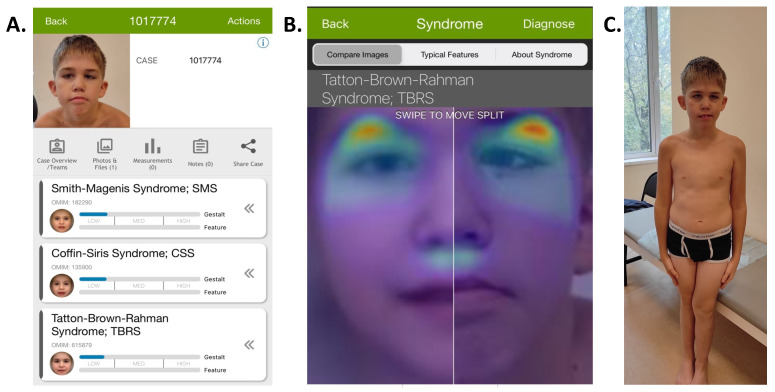
General Face2Gene page, with the Tatton–Brown–Rahman Syndrome in third place with a low gestalt score (**A**). Comparison of heat maps of patient’s image on the left and the reported proband on the right in the Face2Gene system (**B**). The phenotype of the reported proband, with macrocephaly, mild facial dysmorphism, and strabismus (**C**).

Upon objective examination at the age of 10, the proband’s height was 146 cm (+1.10 SD), with a weight of 44 kg (+1.32 SD) and head circumference of 56 cm (+2.23 SD). Some dysmorphic features were noted, including downslanted palpebral fissures; epicanthic fold; convergent strabismus; large, dysplastic, posteriorly rotated ears; a thin upper lip; prominent incisors; and a crowded dental arch (Figure 1A,C). Generalized joint hypermobility was observed. Neurological examination revealed no focal neurological symptoms. No seizures were reported, and the child’s behavior was calm, showing an ability to comprehend directed speech and follow simple instructions. An analysis of the proband’s facial phenotype using a deep learning-based diagnosis assistance system, Face2Gene, did not reveal any high-gestalt match (Figure 1A,B), but TBRS was listed third according to the analysis.

### 3.2. Genetic Analysis

Cytogenetic analysis was conducted on the child with developmental delay, revealing a normal male karyotype of 46, XY. Targeted genetic testing for Martin–Bell syndrome and Beckwith–Wiedemann syndrome was performed using molecular genetic methods, revealing no pathological findings.

**Figure 2:**
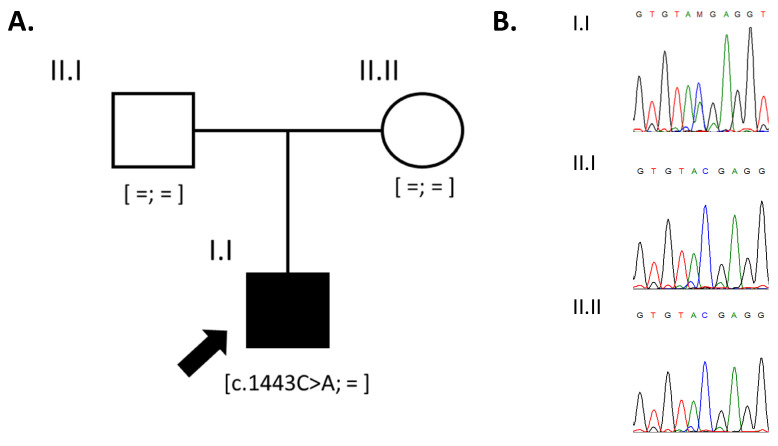
The pedigree of the reported family (**A**) and the results of Sanger sequencing confirming the de novo status of the variant in the *DNMT3A* gene (**B**).

Whole-exome sequencing revealed a previously reported [12] variant in exon 12 in the *DNMT3A* gene (hg19—chr2:25468920G>T) in a heterozygous state. This variant is absent in the gnomAD database and leads to the formation of a premature stop codon—c.1443C>A (p.Tyr481Ter). Segregation analysis of the identified variant (p.Tyr481Ter, NM_175629) in the *DNMT3A* gene confirmed its de novo status (Figure 2B), and based on the ACMG guidelines [13], it was classified as pathogenic (PM2, PVS1, PS2). Heterozygous pathogenic variants in the *DNMT3A* gene are associated with Tatton–Brown–Rahman syndrome, which was confirmed based on the results of the genetic test of the proband.

## 4. Discussion

Our proband presented a mild manifestation of TBRS, prompting a reasonable comparison of their clinical profile with other macrosomic syndromes. Distinguishing these syndromes clinically is feasible and highly important for ensuring the right diagnostic journey. We want to emphasize the importance of using Face2Gene even in patients with mild phenotypes to optimize patients’ diagnostic journey.

In our proband, various traits reminiscent of Sotos syndrome were observed, including accelerated growth alongside normal endocrine function, developmental delays in psychological and motor skills, and behavioral abnormalities featuring elements of aggression and learning difficulties. Notably absent were distinct neurological markers typically associated with Sotos syndrome, such as ataxia, tremor, coordination disturbances, and epileptic activity. It is essential to highlight the divergent growth patterns—Sotos syndrome showcases accelerated growth concurrent with a deficit in body mass, while TBRS patients commonly exhibit obesity. Our patient displayed a trend of rapid weight gain, leading to adherence to a controlled diet over the past 5 months, resulting in a weight reduction of 20 kg. Specific dysmorphic features characteristic of Sotos syndrome, such as dolichocephaly, prominent frontal bosses, macroglossia, and a triangular face, were not observed in our proband [14].

Weaver syndrome is another syndrome that enables one to make a diagnosis different from TBRS. Like TBRS and Sotos syndrome, Weaver syndrome is characterized by the presence of pre- and postnatal overgrowth and variable intellectual disability. However, specific facial features, such as a broad forehead, widely spaced eyes, and almond-shaped palpebral fissures, in combination with a hoarse, low-pitched cry in infants; camptodactyly; and doughy skin, can help to distinguish it from previously described syndromes [15].

Also, both pre- and postnatal macrosomia were noted in our patient, as observed in Beckwith–Wiedemann syndrome (BWS). However, distinctive clinical features, such as visceromegaly, macroglossia, omphalocele, and neonatal hypoglycemia, characteristic of BWS were absent in our proband. It is worth noting that developmental delay is not a typical feature of Beckwith–Wiedemann syndrome [16]. We want to emphasize the specific molecular mechanisms of Beckwith–Wiedemann syndrome, like the abnormal methylation pattern at 11p15.5 or copy number variant of chromosome 11p15.5, which could not be detected by single-gene or multigene NGS panels testing, unlike with TBRS, Sotos syndrome, and Weaver syndrome.

Distinguishing TBRS from Cowden syndrome is facilitated by the absence of multiple hamartomas. However, discerning between the allelic variant of Cowden syndrome—macrocephaly and autism syndrome—and TBRS is more complex. Our patient exhibits partial optic nerve atrophy, a feature not typical of other syndromes involving macrocephaly or excessive growth [17].

Considering the patient’s medical history, including obesity, protruding incisors, and optic nerve atrophy, resembling features found in Cohen syndrome [18], a comprehensive differential diagnosis should encompass Cohen syndrome as well. Unlike our proband and other individuals with TBRS, Cohen syndrome typically presents with low birth weight, postnatal growth delay associated with somatotropic hormone deficiency, and microcephaly.

Several macrosomic syndromes, including TBRS, demonstrate an elevated predisposition to oncological conditions, typified by Sotos syndrome, Beckwith–Wiedemann syndrome, Weawer syndrome, and Cowden syndrome [15,16,17,18,19,20]. The potential association between the mutated genes implicated in growth regulation and an increased susceptibility to tumors warrants careful attention and management, especially following positive results from molecular diagnostics.

## 5. Conclusions

We have presented a genotype–genetic description of a patient with mild Tatton–Brown–Rahman syndrome. Establishing an accurate diagnosis is crucial not only for facilitating medical–genetic counseling and the optimal diagnostic journey, but also for determining the optimal management and surveillance strategy for the patient due to the risk of malignancies.

## Data Availability

The data presented in this study are available on request from the corresponding author.
